# Role of the nigrosome 1 absence as a biomarker in amyotrophic lateral sclerosis

**DOI:** 10.1007/s00415-021-10729-w

**Published:** 2021-08-11

**Authors:** María Isabel Moreno-Gambín, José I. Tembl, Miguel Mazón, Antonio José Cañada-Martínez, Luis Martí-Bonmatí, Teresa Sevilla, Juan F. Vázquez-Costa

**Affiliations:** 1 Neurosonology Laboratory, Department of Neurology, Hospital Universitario y Politécnico La Fe, Valencia, Spain; 2 Radiology and Biomedical Imaging Research Group (GIBI230), Hospital Universitario y Politécnico La Fe, Valencia, Spain; 3 Biostatistics Unit, Instituto de Investigación Sanitaria la Fe (IIS La Fe), Valencia, Spain; 4 ALS Unit, Department of Neurology, Hospital Universitario y Politécnico La Fe, Valencia, Spain; 5 Neuromuscular Research Unit, Instituto de Investigación Sanitaria La Fe (IIS La Fe), Valencia, Spain; 6 Centro de Investigación Biomédica en Red de Enfermedades Raras (CIBERER), Valencia, Spain; 7 Department of Medicine, University of Valencia, Valencia, Spain

**Keywords:** Amyotrophic lateral sclerosis, Primary lateral sclerosis, Progressive muscular atrophy, Hyperechogenicity of *substantia nigra*, Ultrasound, Nigrosome 1

## Abstract

**Introduction:**

The absence of nigrosome 1 on brain MRI and the hyperechogenicity of *substantia nigra* (SNh) by transcranial sonography are two useful biomarkers in the diagnosis of parkinsonisms. We aimed to evaluate the absence of nigrosome 1 in amyotrophic lateral sclerosis (ALS) and to address its meaning.

**Methods:**

136 ALS patients were recruited, including 16 progressive muscular atrophy (PMA) and 22 primary lateral sclerosis (PLS) patients. The SNh area was measured planimetrically by standard protocols. The nigrosome 1 status was qualitatively assessed by two blind evaluators in susceptibility weight images of 3T MRI. Demographic and clinical data were collected and the *C9ORF72* expansion was tested in all patients.

**Results:**

Nigrosome 1 was absent in 30% of ALS patients (36% of PLS, 29% of classical ALS and 19% of PMA patients). There was no relationship between radiological and clinical laterality, nor between nigrosome 1 and SNh area. Male sex (OR = 3.63 [1.51, 9.38], *p* = 0.005) and a higher upper motor neuron (UMN) score (OR = 1.10 [1.02, 1.2], *p* = 0.022) were independently associated to nigrosome 1 absence, which also was an independent marker of poor survival (HR = 1.79 [1.3, 2.8], *p* = 0.013).

**Conclusion:**

In ALS patients, the absence of nigrosome 1 is associated with male sex, UMN impairment and shorter survival. This suggests that constitutional factors and the degree of pyramidal involvement are related to the substantia nigra involvement in ALS. Thus, nigrosome 1 could be a marker of a multisystem degeneration, which in turn associates to poor prognosis.

## Introduction

Amyotrophic lateral sclerosis (ALS) is a neurodegenerative disease clinically characterized by a progressive weakness and signs of upper (UMN) and lower motor neuron (LMN) impairment. According to the degree of UMN and LMN involvement, three phenotypes can be distinguished: classical ALS (cALS), primary lateral sclerosis (PLS) and progressive muscular atrophy (PMA) [1]⁠.

The hallmark of ALS is the presence of TDP-43 aggregates in degenerating UMN and LMN [1]. However, these aggregates can be found far beyond motor neurons in many ALS patients, including *substantia nigra* (SN) [2–4], probably accounting for the extra-motor features seen in ALS patients [1].

By means of transcranial sonography (TCS), a larger area of hyperechogenicity in SN (SNh) has been described in patients with several neurodegenerative diseases, including Parkinson’s disease (PD), atypical parkinsonisms, and ALS [5–14]. In them, the SNh has been proposed to be a marker of vulnerability for neuronal degeneration, probably caused by a disturbance in iron metabolism [10, 12, 15, 16].

Two different parts can be distinguished in the SN in terms of anatomy and function: the pars reticulata (SNpr) and the pars compacta (SNpc). The latter contains dopaminergic neurons, some of which are packed in clusters called nigrosomes [17–19]. In PD patients, postmortem studies have shown that dopaminergic neuronal loss typically affects nigrosomes, especially the largest one, called nigrosome 1 [20]. Nigrosome 1 is located along the rostro-caudal axis of the SNpc, in its dorsal part, and is visible as hyper-intense ovoid sub-structure within the dorsal hypo-intense midbrain region, medial to the cerebral peduncles in high-resolution susceptibility weight (SW) images of 3T and 7T brain magnetic resonance imaging (MRI) [20, 21].

Interestingly, the loss of nigrosome 1 has been demonstrated in 7T and 3T brain MRI of PD patients with postmortem confirmation and has been suggested to be a consequence of neuromelanin loss, an increase in iron content and/or a change in iron oxidation state, associated to the degeneration of dopaminergic neurons of the SNpc [20]. Moreover, the absence of nigrosome 1 differentiates with high-specificity and -sensitivity healthy controls from PD patients [22], where it has been associated with the disease severity and clinical laterality of symptoms [23].

Despite clinical, imaging and neuropathological data suggesting the impairment of SNpc, at least in a subset of ALS patients [2–4], no previous study has assessed nigrosome 1 status in ALS patients.

Therefore, the aims of this study were: (1) to evaluate the nigrosome 1 status in patients with ALS; (2) to evaluate the association between nigrosome 1 status and SNh; (3) to address the contribution of demographical, clinical and genetic factors to the absence or presence of nigrosome 1 in patients with ALS and finally; (4) to evaluate the prognostic value of the absence of nigrosome 1 in the disease.

## Methods

### Subjects and definitions

For this cross-sectional study, patients diagnosed with classical ALS (cALS), PMA or PLS who came to our ALS Unit between February 2014 and June 2019 and gave written informed consent, were recruited. Patients are routinely evaluated by the same neurologist (JFVC), and demographical and clinical data are prospectively recorded in a database. The cALS patients met the El Escorial revised criteria of possible, probable or definitive ALS [24]. PMA was defined as a progressive isolated impairment of LMN in at least two regions [25], and PLS as a progressive isolated impairment of UMN in at least one region other than the lumbar region [26].

Patients were followed up until October 2020 and the date of death or tracheostomy were recorded.

### Genetic analysis

All patients were screened for *C9ORF72* with repeat primed PCR, as previously reported [27].

### Clinical and analytical variables

Age, gender, time of symptom onset, region of onset (bulbar vs. spinal) and King’s clinical staging were recorded for all patients.

ALS patients were examined at the time of recruitment by the same expert neurologist (JFVC). The disability (measured with the ALSFRS-R score) [28] and degree of UMN impairment (measured with the UMN score, for a maximum of 16) [29], were recorded. The laterality of symptoms was established as the side of onset or, in bulba onset patients, of clinical predominance. Parkinsonian features (such as bradykinesia and cogwheel rigidity) were not systematically recorded, since they may be masked by and/or confounded with UMN and LMN signs [30].

The presence of behavioral and language disturbances was systematically assessed and the diagnosis of ALS with frontotemporal dementia (ALSFTD) was established based on the current criteria [31].

### Nigrosome 1 examination

MR imaging examinations were performed in a 3T scanner (Signa HDxt, GE Healthcare, Milwaukee, WI, USA) using transmit–receive head coil array with eight elements. SW images were obtained with a 3D multi-echo gradient echo T2*-weighted sequence acquired on the transverse plane (TR = 43 ms; 9 echoes with center TE around 26 ms; TE = 15.1–35.8 ms with 5.1 echo spacing; FOV = 220 × 220 mm; matrix = 256 × 256 mm; slice thickness = 2 mm, flip angle = 15°; bandwidth = 62.5 Hz/px).

**Fig. 1:**
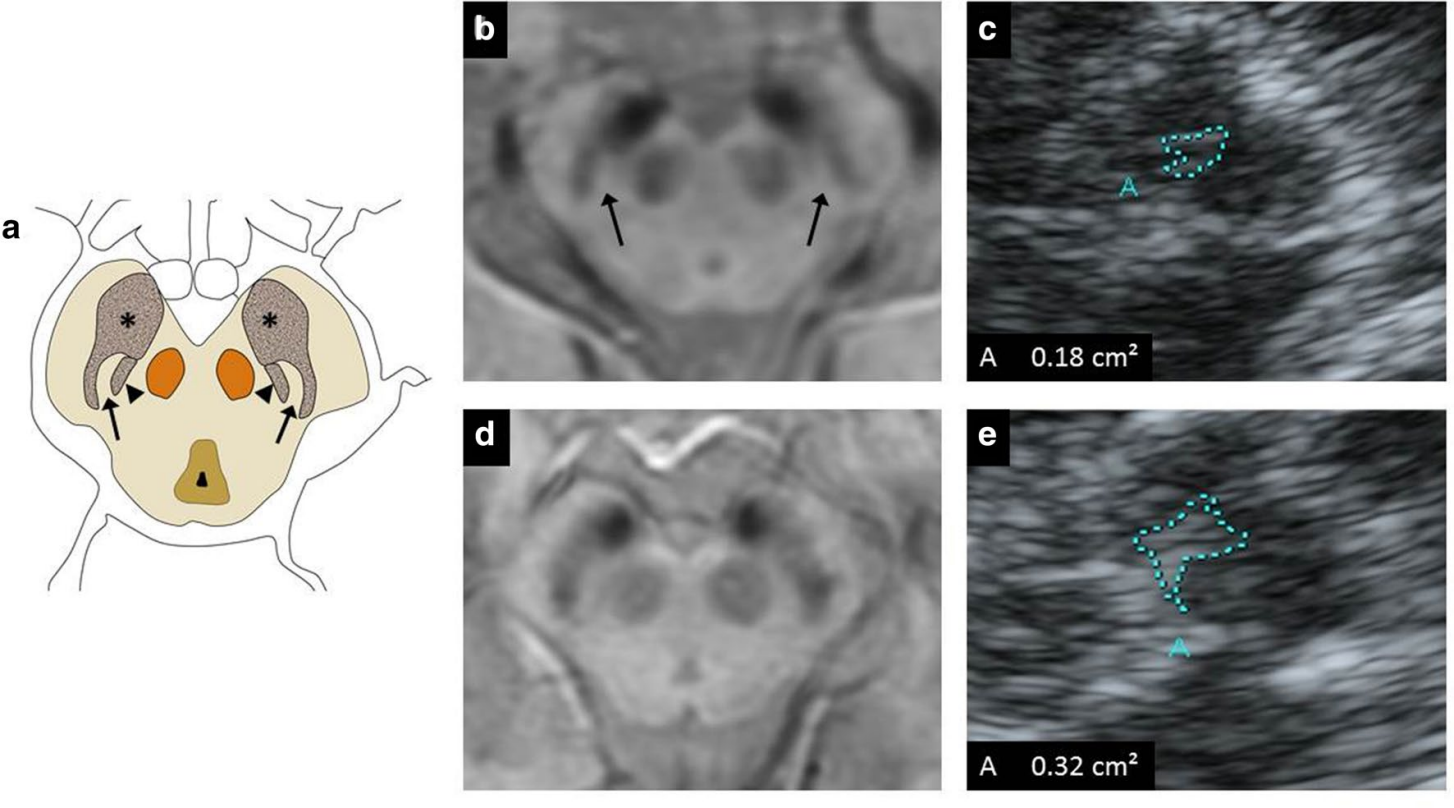
Anatomic representation of mesencephalon including nigrosome 1 and its surrounding structures (**a**, *asterisk* = substantia nigra; *arrowhead* = medial lemniscus; *arrow* = nigrosome 1; *circle* = red nucleus). Nigrosome 1 present on susceptibility weight images of MRI (**b**, *arrow*) and area of the SNh (**c**). Nigrosome 1 absent on susceptibility weight images of MRI (**d**) and larger area of SNh (**e**). ALS: amyotrophic lateral sclerosis; SNh: substantia nigra hyperechogenicity

Nigrosome 1 status on brain MRI was visually assessed by a neurologist (MIMG) with 4 years of experience and one expert neuroradiologist (MMM), blinded to each other and to the patients’ data. For the nigrosome 1 evaluation, axial brain MRI SWI sequences at the midbrain level were selected. Healthy nigrosome 1 is a comma-shaped structure located in the posterior area of the SN. It has a high signal on SWI sequences compared to the surrounding elements: the SNpr, anterior and laterally; and the medial lemniscus, medially. Healthy nigrosome 1 and its surrounding structures has been described to resemble a “swallow tail” (Fig. 1) [21].

Nigrosome 1 status was qualitatively assessed as (Fig. 1): present (“swallow tail” identified), absent (“swallow tail” not identified) or doubtful (when the quality of the images did not allow to define the nigrosome 1 status). Discrepancies between the two readers were resolved by consensus. Patients with doubtful nigrosomes (3 of all patients), in whom a consensus on nigrosome status was not reached, were excluded from further analysis.

### TCS examination

In most patients, a TCS examination was performed within 3 months from MRI, by an expert neurologist sonographer (JIT), who was blind to the clinical data, with the same ultrasound system (Toshiba Aplio XG, Tokyo, Japan 2008) equipped with a 2.5 MHz phased-array transducer to obtain B-mode images through a temporal acoustic bone window. Right and left SNh were obtained and measured by this examiner, as previously reported [32] (Fig. 1).

### Statistical analysis

Data were summarized by the mean, standard deviation, median and the first and third quartiles for the continuous variables, and by the relative and absolute frequencies for the categorical variables. For the statistical analysis, the nigrosome was considered to be present in a patient when both nigrosomes were visible, and absent, when either of both nigrosomes was absent. Inter- and intra-rater agreements were assessed using weighted κ test for the ratings (present, absent, doubtful). To assess the agreement between the side of disease onset and the absence of contralateral nigrosome, a Cohen κ test was performed. A mixed logistic regression model, including the subject and side as a random effect, was performed to compare the SNh area according to the nigrosome status. The association of demographical and clinical variables of ALS patients with the absence of nigrosome was assessed by a logistic regression model. The effect of the absence of nigrosome in survival was assessed with a Cox regression model and the results are displayed in a forest plot. Expected survival curves for subpopulations according to nigrosome absence, were calculated based on Cox model and are represented in a Kaplan–Meier curve (Fig. 3).

In both models, co-variables were selected based on previous literature and pre-specified hypothesis. *P* values of < 0.05 were considered statistically significant. All the statistical analyses and graphs were performed using version 4.3.0 of the R software.

## Results

### Population characteristics

One hundred and thirty six ALS patients (72% cALS; 12% PMA and 16% PLS) were recruited for this study. Briefly, mean age of patients was 62 years, and 56% were male. Most patients had a spinal onset of symptoms (70%), 15% were diagnosed with concomitant FTD and 12% carried a *C9ORF72* expansion. At the time of examination, median disease duration was 17 months, and patients presented a mean ALSFRS-R of 37 and a median progression rate of 0.6. The demographic, clinical, and genetic characteristics of ALS patients, as per phenotype, are summarized in Table 1.

**Table 1:** Demographic, clinical, and genetic characteristics of ALS patients as per phenotype

	PMA (*n* = 16)	cALS (*n* = 98)	PLS (*n* = 22)
Age (years)			
Mean (SD)/*n* (%)	62.33 (13.87)	61.94 (11.3)	64.6 (8.92)
Median (IQR)	64.8 (58.53, 70.57)	62.97 (55.66, 70.56)	65.98 (59.43, 70.35)
Gender (male)			
*n* (%)	13 (81.25%)	50 (51.02%)	13 (59.09%)
FTD			
*n* (%)	0 (0%)	18 (18.37%)	2 (9.09%)
Region of onset			
Bulbar *n* (%)	1 (6.25%)	33 (33.67%)	7 (31.82%)
Spinal *n* (%)	15 (93.75%)	65 (66.33%)	15 (68.18%)
Disease duration (months)			
Mean (SD)/*n* (%)	49.26 (47.17)	20.74 (19.95)	75.64 (67.05)
Median (IQR)	36.47 (13.72, 54.97)	13.87 (8.48, 24.49)	54.57 (35.3, 93.92)
King’s clinical stage			
1 *n* (%)	5 (31.25%)	6 (6.12%)	2 (9.09%)
2 *n* (%)	10 (62.5%)	62 (63.27%)	6 (27.27%)
3 *n* (%)	0 (0%)	27 (27.55%)	12 (54.55%)
4 *n* (%)	1 (6.25%)	3 (3.06%)	2 (9.09%)
ALSFRS-R			
Mean (SD)/*n* (%)	40.38 (3.69)	37.37 (6.2)	33.52 (7.92)
Median (IQR)	40.5 (38.75, 43)	39 (34, 42)	35 (30, 38)
UMN score			
Mean (SD)/*n* (%)	0.5 (1.32)	5.42 (5.07)	12 (5.49)
Median (IQR)	0 (0, 0)	4 (1, 9)	13 (9, 14)
Progression rate			
Mean (SD)/*n* (%)	0.35 (0.41)	0.92 (0.78)	0.38 (0.38)
Median (IQR)	0.2 (0.1, 0.32)	0.76 (0.33, 1.14)	0.24 (0.14, 0.47)
C9ORF72 mutation			
*n* (%)	0 (0%)	15 (15.31%)	1 (4.55%)

*ALS* amyotrophic lateral sclerosis, *ALSFRS-R* Revised Amyotrophic Lateral Sclerosis Functional Rating Scale, *cALS* classical ALS, *FTD* frontotemporal dementia, *PLS* primary lateral sclerosis, *PMA* progressive muscular atrophy, *UMN-score* Upper Motor Neuron Score

### Contribution of demographic, clinical and genetic variables to nigrosome in the ALS patients

The nigrosome 1 status was assessable in 133 (97.7%) patients. The intra- and inter-rater agreement of the nigrosome 1 status was acceptable (weighted *κ* of 0.6 and 0.69, respectively).

At least one nigrosome 1 was absent in 30% (*n* = 39) of ALS patients, being more frequent in PLS patients (36%), followed by cALS patients (29%) and PMA patients (19%). Nigrosome 1 was absent in both sides in most patients (63%), only in the left side in 14% and in the right side in 20% of patients. There was no agreement between radiological laterality and the side of symptoms onset (Cohen *κ* = 0.03). Table 2 summarizes patients’ characteristics according to the nigrosome 1 status. Overall, patients with absent nigrosome 1 were more frequently male, had higher UMN score, and were more frequently diagnosed with FTD and with a *C9ORF72* expansion. Interestingly, they showed similar ALSFRS-R score than patients with both nigrosomes 1, despite presenting shorter disease duration. Accordingly, they showed somewhat faster disease progression rate.

**Table 2:** Demographic, clinical, and genetic characteristics of ALS patients according to nigrosome 1 status

	Nigrosome 1 absent (*n* = 39)	Nigrosome 1 present (*n* = 94)	Nigrosome 1 doubtful (*n* = 3)
Age (years)			
Mean (SD)/*n* (%)	63.29 (9.1)	61.96 (11.99)	65.44 (15.62)
Median (IQR)	64.81 (56.36, 69.94)	63.01 (56.18, 70.56)	73.76 (60.59, 74.45)
Male gender			
*n* (%)	28 (71.79%)	46 (48.94%)	2 (66.67%)
FTD			
n (%)	9 (23.08%)	11 (11.7%)	0 (0%)
Region of onset			
Bulbar *n* (%)	14 (35.9%)	26 (27.66%)	1 (33.33%)
Spinal *n* (%)	25 (64.1%)	68 (72.34%)	2 (66.67%)
C9ORF72 mutation			
*n* (%)	7 (17.95%)	9 (9.57%)	0 (0%)
Time from onset (months)			
Mean (SD)/*n* (%)	25.12 (22.42)	36.71 (46.56)	17.94 (10.73)
Median (IQR)	15.73 (11.63, 33.38)	17.22 (8.89, 47.66)	19.3 (12.95, 23.62)
ALSFRS-R			
Mean (SD)/*n* (%)	36.89 (5.74)	37.21 (6.88)	37.33 (2.08)
Median (IQR)	37.5 (35, 41)	39 (34, 42)	38 (36.5, 38.5)
UMN Score			
Mean (SD)/*n* (%)	7.47 (5.45)	5.28 (5.78)	3.67 (4.73)
Median (IQR)	7.5 (2, 13)	3 (1, 9)	2 (1, 5.5)
Progression rate			
Mean (SD)/*n* (%)	0.86 (0.8)	0.73 (0.71)	0.94 (0.75)
Median (IQR)	0.68 (0.35, 1)	0.44 (0.2, 1)	0.72 (0.52, 1.25)

*ALS* amyotrophic lateral sclerosis, *ALSFRS-R* Revised Amyotrophic Lateral Sclerosis Functional Rating Scale, *cALS* classical ALS, *FTD* frontotemporal dementia, *PLS* primary lateral sclerosis, *PMA* progressive muscular atrophy, *UMN-score* Upper Motor Neuron Score

**Fig. 2:**
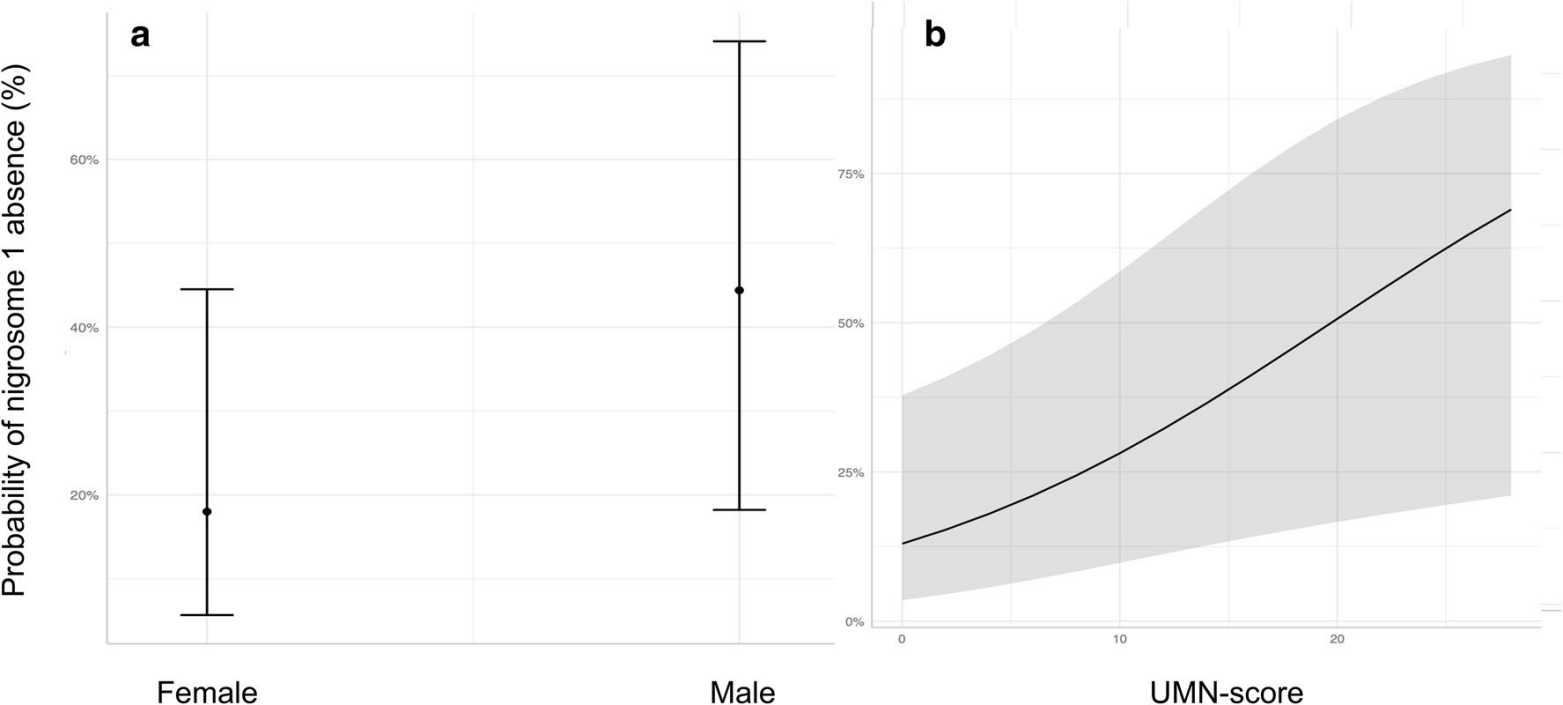
Graphical representation of the estimated effect of male sex (**a**) and upper motor neuron score (**b**) in the absence of nigrosome 1, as per the multivariable model

The multivariable model showed an independent association between the absence of nigrosome 1 and male gender (estimate = 3.637, *p* = 0.005) and higher UMN-score (estimate = 1.101, *p* = 0.022). Conversely, no statistically significant differences in ALSFS-R score, FTD diagnosis, C9ORF72 status and disease duration were found (see Table 3 and Fig. 2).

**Table 3:** Multivariable model assessing the association between the absence of nigrosome 1 and demographic, clinical and genetic characteristics of ALS patients

	OR	Lower 95	Upper 95	*p*
Age	1.021	0.982	1.065	0.304
Male sex	**3.637**	**1.507**	**9.38**	**0.005**
FTD	1.657	0.551	4.859	0.358
UMN-score	**1.101**	**1.016**	**1.2**	**0.022**
Disease duration	0.985	0.967	0.998	0.056
ALSFRS-R	1.003	0.933	1.085	0.933
C9ORF72 mutation	1.845	0.532	6.263	0.324

*ALSFRS-R* Revised Amyotrophic Lateral Sclerosis Functional Rating Scale, *FTD* Fronto Temporal Dementia, *UMN-score* Upper Motor Neuron Score. In bold, statistically significant results

### Nigrosome and prognosis

**Fig. 3:**
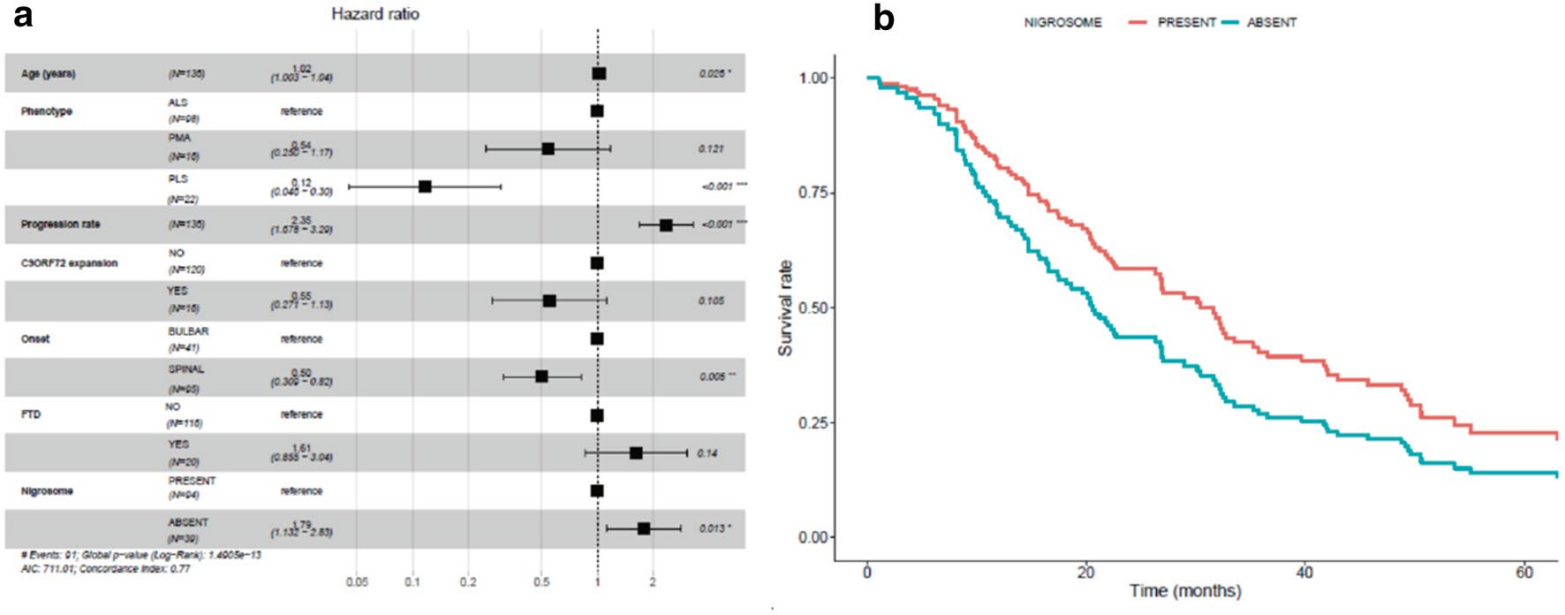
Forest plot displaying the effect (Hazard Ratio) of co-variables in the survival of ALS patients (**a**). Kaplan–Meier curve, based on the Cox model, representing the independent effect of nigrosome 1 absence in the survival of ALS patients (**b**). Red = Nigrosome 1 present; Blue = Nigrosome 1 absent. Time is measured in months. *FTD* frontotemporal dementia, *PLS* primary lateral sclerosis, *PMA* progressive muscular atrophy

In the Cox model, the absence of nigrosome 1 was found to be an independent predictor of poor prognosis in ALS patients (HR = 1.79; *p* value = 0.013) together with age (HR = 1.02; *p* value = 0.025) and progression rate (HR = 2.35; *p* value < 0.001), while spinal onset (HR = 0.50; *p* value = 0.005) and PLS phenotype (HR = 0.12; *p* value < 0.001) were protective factors (Fig. 3).

### Nigrosome and SNh area

TCS was performed in 111 patients within 3 months from MRI (median delay of 1 day), and it was possible to measure the SNh area through the temporal acoustic bone window in 95 (81.9%) of them.

**Fig. 4:**
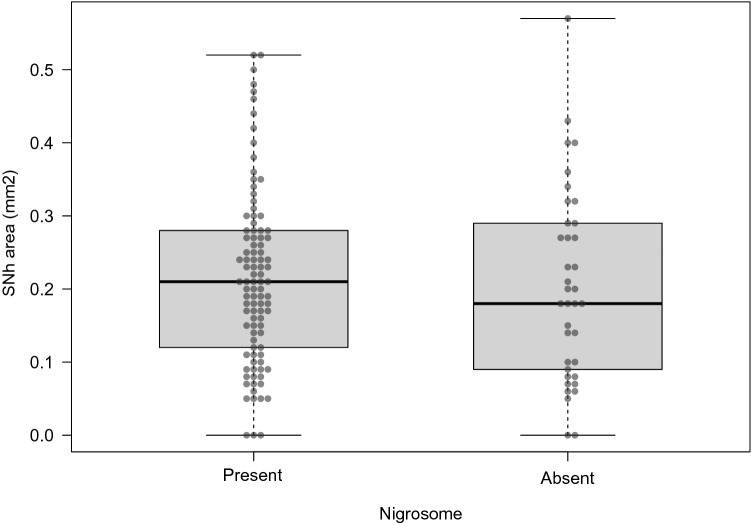
Box plots representing the SNh area according to the nigrosome status. SNh: substantia nigra hyperechogenicity

No differences in the SNh area were found according to the nigrosome status (OR = 0.46, *p* = 0.63, Fig. 4).

## Discussion

The SNpc is a small midbrain region comprising neuro-melanin-containing dopaminergic neurons (some of them packed in nigrosomes), which becomes frequently affected in neurodegenerative diseases with motor involvement, such as parkinsonisms and motor neuron diseases [2, 4, 10]. Up till now, several imaging biomarkers have been described in the SN in these diseases. The SNh, measured by TCS, is thought to reflect iron deposits and has been shown to be a marker of SN vulnerability rather than neuronal loss [10, 16]. Accordingly, the SNh is not related with motor symptoms, but with genetic and constitutional factors that increase the risk of those neurodegenerative diseases [10, 16]. Unlike the SNh, the absence of nigrosome 1, detected in high SWI of brain MRI, is thought to represent a neuro-melanin/iron imbalance due to neuronal loss, and has been associated with motor symptoms in the contralateral brain hemisphere in PD patients [20, 23]. Despite the profuse literature about both biomarkers, to the best of our knowledge, no previous study has compared the absence of nigrosome 1 with the SNh in any neurodegenerative disease. Here, we demonstrate that there is not an association between the SNh and the absence of nigrosome 1, reinforcing the idea that they measure different pathophysiological processes. In short, the SNh probably measures an increase in iron content in the SNpc acting as a marker of neuronal vulnerability, while the nigrosome 1 absence is rather a marker of neuro-melanin loss in the SNpc. Considering this, it is not surprising that, in some diseases specifically causing SNpc degeneration (such as PD), the SNh is as frequently found as the nigrosome 1 absence [22]. Conversely, in other diseases, such as ALS, the SNh is much more frequently found than the absence nigrosome 1 on MRI or on the neuropathological assessment.

Thus, in postmortem studies, the SNpc becomes involved in about 20–30% of patients [2, 4, 33], a similar proportion to the nigrosome 1 loss found in our study. Different neuropathological patterns of involvement of the SNpc can be distinguished among ALS cases. On the one hand, the SNpc involvement is a feature of a subset of cALS patients being in an advanced neuropathological stage (2–4) [2, 4]. On the other hand, the SNpc involvement is a hallmark of ALS with pallidonigroluysian degeneration [2, 4, 33]. Similarly, different clinical patterns of patients combining motor neuron disease and parkinsonism have been described (e.g. ALS-parkinsonism, PLS-parkinsonism, ALS-FTD-parkinsonism…) [34]. Moreover, parkinsonian features and a dopaminergic deficit have also been described in the striatum of a variable proportion of cALS patients [30, 35–37]. Intriguingly, no association between extrapyramidal features and dopaminergic striatal deficit in ALS patients has been found [30, 35, 36], suggesting that other circuits are responsible of these symptoms. Accordingly, the SNh was not associated with clinical features in ALS patients, but with genetic and constitutional factors [16]. However, SNh is not a marker of neuronal loss and, up till now, no previous study has assessed nigrosome 1 in ALS patients.

In this study, we show for the first time that nigrosome 1 is absent, on at least one side of the *SNpc,* in 30% of ALS patients with different phenotypes, compared to about 95% of PD patients and 10% of healthy controls [22]. Interestingly, the neuropathologic involvement of the SNpc has been found in a similar percentage of patients in postmortem studies [2, 4, 33].

Unlike in PD patients [23], in most ALS patients, the nigrosome 1 was absent in both sides and, in those with an asymmetric involvement, no relationship with the clinical side of onset was found. Moreover, no associations between nigrosome 1 absence and age, disease duration or disability were found. Furthermore, although the frequency of FTD and *C9ORF72* mutations was proportionally higher in patients with absent nigrosome 1, no independent association could be found in the multivariable model (perhaps due to the small sample size). However, male sex and the UMN score (a proxy of the degree of UMN impairment) were independently associated with the nigrosome 1 absence. Moreover, the nigrosome 1 absence was most frequent in PLS patients and least frequent in PMA patients. These findings are not surprising, considering previous literature. Firstly, male sex is a well-known risk factor for ALS, Parkinson’s disease and larger area of hyperechogenicity of *substantia nigra* [16]. Second, both parkinsonian features and dopaminergic dysfunction have been more frequently described in male and UMN-predominant ALS patients [30, 35]. Third, extrapyramidal features are much more frequent in PLS than in ALS patients and can respond to levodopa [38]. All these suggest a relationship between male sex, pyramidal and extrapyramidal involvement. The distinction between extrapyramidal and pyramidal symptoms and signs is complex and it has been hypothesized that parkinsonian traits in UMN-predominant ALS patients could actually be attributed to spasticity [30]. However, our data showing an association between UMN signs and the absence of nigrosome 1, rather suggest a direct relationship between the degree of pyramidal and extrapyramidal involvement in ALS patients. This fits well with the proposed model of corticofugal axonal spread of the disease [39] and the existence of a direct cortico-nigral pathway [40]. Thus, the absence of nigrosome 1 in ALS patients could be a marker of the cerebral neuropathological expansion of the disease to the second neuropathological stage, as proposed by Brettschneider et al. [2]. In this respect, the lack of association between the nigrosome absence and disability or disease duration is not surprising, since the neuropathological extension in ALS is not related with none of these variables [2, 3, 33].

Finally, we found that the nigrosome 1 absence is an independent prognostic factor, when adjusted for other well-known risk factors. As commented above, the absence of nigrosome 1 could be a marker of neuropathological multisystem degeneration, which ultimately associates to poor prognosis [3], as found in ALS patients with concomitant FTD or other extramotor symptoms [31, 41].

### Strengths and limitations

Our study represents the first study assessing the status of nigrosome 1 and SNh in a large cohort of thoroughly characterized ALS patients, which allowed the use of multivariable analysis.

The main limitation is that parkinsonian features were not assessed systematically and consequently have not been analyzed. However, interpreting parkinsonian features in ALS patients may be challenging, since they can be masked by UMN and LMN signs [30]. Moreover, previous studies have not found relationship between extrapyramidal traits and SN dysfunction [30, 35, 36]. Another limitation is that the assessment of the nigrosome 1 is observer-dependent. To minimize this limitation, the nigrosome 1 was assessed by two experienced readers and discrepancies between the two were resolved by consensus. The use of 123 I-FP-CIT scintigraphy could have help to validate our results of nigrosome 1 absence, but was not available in this study. Although we studied a large cohort of ALS patients, some characteristics (FTD, C9ORF72 expansion) are infrequent, which could have limited the power to detect an association between nigrosome 1 and those variables. Finally, healthy controls or ALS mimics were not included to assess its role as a diagnostic biomarker. However, given its frequency in ALS patients, it does not appear to be useful in the diagnostic work up of ALS patients.

## Conclusion

The absence of nigrosome 1 is found in up to a third of ALS patients and is not related with the SNh area. Moreover, it is independently associated with male sex, UMN impairment and shorter survival. This suggests that constitutional factors and the degree of pyramidal impairment influence the neuropathological involvement of the SNpc in ALS patients. Thus, nigrosome 1 could be a marker of a multisystem degeneration, which in turn associates to poor prognosis. Neuropathological studies are warranted to confirm our findings.

## Data Availability

The data that support the findings of this study are available from the corresponding author, (JFVC), upon reasonable request. There are no additional unpublished data from the study.
